# MTGR1 is required to maintain small intestinal stem cell populations

**DOI:** 10.21203/rs.3.rs-3315071/v1

**Published:** 2023-09-21

**Authors:** Christopher Williams, Rachel Brown, Yue Zhao, Jing Wang, Zhengyi Chen, Koral Blunt, Jennifer Pilat, Bobak Parang, Yash Choksi, Ken Lau, Scott Hiebert, Sarah Short, Justin Jacobse, Yanwen Xu, Yilin Yang, Jeremy Goettel

**Affiliations:** Vanderbilt University Medical Center; Vanderbilt University; Vanderbilt University; Vanderbilt University Medical Center; Vanderbilt University; Vanderbilt University; Vanderbilt University; Vanderbilt University; Vanderbilt University Medical Center; Vanderbilt University; Vanderbilt University; University of Iowa; Vanderbilt; Vanderbilt; Vanderbilt; Vanderbilt

## Abstract

Undifferentiated intestinal stem cells (ISCs), particularly those marked by *Lgr5*, are crucial for maintaining homeostasis and resolving injury. *Lgr5*+ cells in the crypt base constantly divide, pushing daughter cells upward along the crypt axis, where they differentiate into a variety of specialized cell types. This process requires coordinated execution of complex transcriptional programs, which allow for the maintenance of undifferentiated stem cells while permitting differentiation of the wide array of intestinal cells necessary for homeostasis. Thus, disrupting these programs may negatively impact homeostasis and response to injury. Previously, members of the myeloid translocation gene (MTG) family have been identified as transcriptional co-repressors that regulate stem cell maintenance and differentiation programs in multiple organ systems, including the intestine. One MTG family member, myeloid translocation gene related 1 (MTGR1), has been recognized as a crucial regulator of secretory cell differentiation and response to injury. However, whether MTGR1 contributes to the function of ISCs has not yet been examined. Here, using *Mtgr1*^−/−^ mice, we have assessed the effects of MTGR1 loss on ISC biology and differentiation programs. Interestingly, loss of MTGR1 increased the total number of cells expressing *Lgr5*, the canonical marker of cycling ISCs, suggesting higher overall stem cell numbers. However, expanded transcriptomic analyses revealed MTGR1 loss may instead promote stem cell differentiation into transit-amplifying cells at the expense of cycling ISC populations. Furthermore, *ex vivo* intestinal organoids established from *Mtgr1* null were found nearly completely unable to survive and expand, likely due to aberrant ISC differentiation, suggesting that *Mtgr1* null ISCs were functionally deficient as compared to WT ISCs. Together, these results identify a novel role for MTGR1 in ISC function and suggest that MTGR1 is required to maintain the undifferentiated state.

## Introduction

The intestinal epithelium is under constant metabolic, mechanical, and microbial stress and, as such, is in a continual state of regeneration and renewal. While models for cell function in the gut are evolving, it has been repeatedly demonstrated that intestinal stem cells (ISCs) are key to maintaining intestinal homeostasis and response to injury^1^. Maintenance of intestinal health and regeneration requires the orchestrated execution of stem cell, early progenitor, and differentiation programs, usually via coordinated activation and suppression of transcriptional circuits, to ensure proper stem cell function and balanced downstream lineage allocation^2^.

To accommodate the constant cellular turnover of the intestinal epithelium, ISCs rapidly proliferate in the crypt base, pushing their daughter cells out of the crypt and up the intestinal villus while undergoing terminal differentiation into an assortment of secretory and absorptive cell types. These highly proliferative crypt base ISCs, termed crypt base columnar cells (CBCs), are identified by their expression of the gene, leucine-rich repeat containing G-protein coupled receptor 5 (*Lgr5*)^1^. *Lgr5* expression is highly regulated, most notably by Wnt and Notch signaling pathways, and is suppressed upon exit of the stem cell niche and activation of cellular differentiation programs^3^. Interestingly, while WNT pathway activation in CBCs upregulates *Lgr5* expression, LGR5 protein also functionally promotes Wnt signaling and ISC biology by serving as the receptor for the potent Wnt agonist, R-spondin^4^. As proliferation continues, daughter cells from CBCs move further up the crypt base into the transit amplifying (TA) compartment. Here, cells proliferate even more rapidly than parental CBCs, yet simultaneously begin lineage commitment and terminal differentiation.

In addition to *Lgr5* + CBCs, other populations of ISCs have been identified, many of which are less proliferative than CBCs at homeostasis but are “activated” as part of intestinal regenerative programs. These cell populations are denoted by expression of specific genes, such as *Bmi1, mTert, Hopx, Lrig1*, and *Clu;* often reside higher in the crypt base than CBCs in the + 4/+5 position; and may have begun the process of lineage commitment^5,6^. However, functional compensation by these slowly cycling ISC populations may still require dedifferentiation and interconversion to CBCs, in which ISCs gain expression of *Lgr5* and take up residence in the crypt base. Interestingly, the ability to reconstitute the CBC population has even been noted in committed progenitors of the secretory and absorptive lineages^7–10^. Thus, coordinating ISC function and differentiation among differentiated cell types and different ISC populations is a far from linear process, yet remains a crucial component of both intestinal homeostasis and injury responses.

MTGR1 (*Cbfa2t2*) is a member of the three-protein myeloid translocation gene (MTG) family of transcriptional co-repressors, which were originally identified in translocation fusion proteins driving acute myeloid leukemia^11^. MTGs, also including MTG16 and MTG8, serve as scaffolding proteins that orchestrate the formation of repression complexes containing histone deacetylases, other co-repressor proteins, and DNA binding factors, thereby modifying chromatin state of key loci^12^. In the intestine, MTGs are known to serve important functions, as MTG-deficient mice display a range of unprovoked intestinal phenotypes. For example, loss of MTG8 (*Mtg8*^−/−^) results in deletion of the midgut, while loss of MTG16 or MTGR1 (*Mtg16*^−/−^ and *Mtgr1*^−/−^) alters intestinal proliferation, apoptosis, migration, and lineage specification^13–16^. *Mtgr1*^−/−^ mice are also exquisitely sensitive to dextran sodium sulfate (DSS)-induced injury, with marked depletion of viable, regenerative crypts post-injury^17^. Finally, we have previously identified MTGR1 as a key regulator of intestinal differentiation into the secretory cell fate, as *Mtgr1*^−/−^ mice have greatly reduced numbers of Paneth, goblet, and enteroendocrine cells^15,18^.

Interestingly, work aiming to determine signaling pathways by which MTGs mediate these functions have uncovered roles for MTGs in modulating Wnt and Notch signaling pathways^18,19^. Despite contributions to these key ISC-associated signaling pathways, the exact role of MTGR1 in ISC biology remains incompletely understood. Here, we have assessed the impact of MTGR1 deficiency on stem cell identity and function in the small intestine both *in vivo* and *ex vivo*. Together, these studies uncover a crucial role for MTGR1 in maintaining proper ISC identity and function and expand our knowledge of the mechanisms regulating intestinal differentiation and regeneration.

## Methods and Materials

### Mice

*Mtgr1*^−/−^ mice were previously established and characterized^15^. *Lgr5-EGFP-IRES-creERT2* mice were a generous gift from Dr. Robert Coffey^1^. Male and female age-matched, littermate WT and *Mtgr1*^−/−^ mice were used for all experiments. Mice were cohoused and maintained on standard chow with 12-hour light/dark cycles. All *in vivo* procedures were carried out in accordance with protocols approved by the Vanderbilt Institutional Animal Care and Use Committee.

### Enteroid culture

Enteroids were established from 8–12-week old WT and *Mtgr1*^−/−^ mice as previously described^18,20^. Crypts were counted and equal numbers plated in growth factor reduced Matrigel^®^ (Corning) and overlaid with “ENR” mini-gut culture media. (Advanced Dulbecco’s modified Eagle’s medium–F-12 [Gibco], 100 U/ml penicillin, 100 μg/ml streptomycin, 1X N2 [Gibco], 1X B27 [Gibco], 1X Glutamax [Gibco], 1mM HEPES [Gibco], 20% R-spondin conditioned media [generated from R-spondin-expressing cells generously provided by Dr. Jeff Whitsett, Cincinnati Children’s Hospital], 10% Noggin conditioned media [generated from Noggin-expressing cells generously provided by Dr. G.R. van den Brink, described in ^21^], and 50ng/ml EGF [R&D Systems]). Media at plating was supplemented as noted in the text with various inhibitors, described in **Supplemental Table S1**. To determine plating efficiency, the number of viable enteroids was assessed at day 1 post-plating and normalized to the number of crypts plated. Viability was determined by daily enteroid counts and normalized to the number of enteroids established on day 1. ImageJ software (version 1.51) was used to measure organoid size and count crypt buds.

### Enteroid hMTGR1 addback

GFP and human *MTGR1* were cloned into the pLEX-307 vector (a gift from David Root, Addgene plasmid 41392). pLEX-307-GFP and pLEX-307-*MTGR1* were transfected into HEK 293T cells (ATCC CRL-3216) along with psPAX2 and pMD2.g (gifts from Didier Trono, Addgene plasmids 12260 and 12259). After 48 hours, supernatants were collected and viral particles were concentrated by overnight centrifugation at 9,500g at 4°C. Pelleted lentiviral particles were resuspended in mouse Intesticult media (StemCell Technologies) supplemented with 10μM Y-27632 (Tocris) and mixed with duodenal crypt isolations from WT or *Mtgr1*^−/−^ mice. Crypt/virus mixtures were incubated for 2 hours at 37°C prior to washing and plating in Matrigel plugs overlaid with ENR media supplemented with CHIR 99021 (3μM, Tocris) and Y-27632. After 4 days, CHIR 99021 and Y-27632 were removed. Viability and gene expression were assessed at day 7 post-plating.

### Immunohistochemistry

For *in vivo* analysis, mice were sacrificed and intestinal tissue was “Swiss-rolled” prior to fixation in 10% neutral-buffered formalin. Intestinal samples were then paraffin-embedded and 5 μm sections were cut by the Vanderbilt Translational Pathology Shared Resource (TPSR). For enteroid staining, cultures were collected and fixed as described previously^22^. Briefly, enteroids were collected and fixed in 10% neutral buffered formalin on ice for 20 min. After washing, the enteroid pellet was resuspended in 2% agarose dissolved in PBS. The agarose was allowed to solidify for 10 min, placed in 70% ethanol, and paraffin-embedded.

For all fluorescent immunohistochemistry (IHC), antigen retrieval consisted of 10 minutes boiling in 10 mM sodium citrate (pH 6) prior to blocking. After blocking, samples were incubated with antibodies against E-cadherin (BD Biosciences, 1:500), Ki67 (Abcam, 1:1000), phospho-histone H3 (Millipore, 1:400), cleaved caspase-3 (Cell Signaling Technology, 1:400), or β-catenin (BD Biosciences, 1:500) overnight at 4°C. Secondary antibodies conjugated to 488 or 594 Alexafluor dyes (Invitrogen) were incubated for 2 hours at room temperature. Nuclear staining was done with ProLong Gold antifade reagent with DAPI (Invitrogen). Staining was visualized with a Nikon Eclipse E800 microscope and Zyla SCMOS camera. Images were processed and quantified using Nikon NIS-Elements Basic Research software.

### qRT-PCR analysis

Freshly isolated murine small intestinal crypts were collected and homogenized in TRIzol reagent (Thermo Fisher) using a 21g needle. RNA was isolated using the Rneasy Mini Kit (Qiagen) with on-column DNAse digestion. cDNA was synthesized using the qScript cDNA synthesis kit (Quantabio). qPCR reactions were run using PerfeCTa SYBR Green SuperMix ROX (Quantabio) and primers designated in **Supplemental Table S2**. For analysis of human *MTGR1*, probes for *MTGR1* (Hs00602520_m1, Thermo Fisher) and *Gapdh* (Mm99999915_g1, Thermo Fisher) were used in conjunction with TaqMan Universal Master Mix II (Thermo Fisher). All samples were run in triplicate and target gene expression was analyzed using the delta–delta Ct method normalized to *GAPDH*.

### RNA Scope

High-resolution RNA *in situ* hybridization was performed using the RNAscope^®^ Multiplex Fluorescent V2 assay or RNAscope^®^ 2.5HD Assay – Brown, according to the manufacturer’s instructions (ACDBio). Antigen retrieval was performed under standard pretreatment conditions as specified by the manufacturer. Probes were directed against mouse *Cbfa2t2* (#434601), *Lgr5* (#312171), or *Clu* (#427891). Fluorescent assay samples were mounted with ProLong Gold antifade reagent with DAPI and imaged as described above.

### RNA-sequencing

For RNA-sequencing, small intestine crypts were isolated from 3 WT and 3 *Mtgr1*^−/−^ mice. Following crypt isolation, a portion of the samples were collected and homogenized in TRIzol reagent while the remaining crypts were plated for enteroid culture. After 24 hours, half of the plated enteroids were collected for RNA extraction, while the remaining enteroids were cultured for an additional 48 hours and harvested at 72 hours post-plating. Additional samples were collected from passaged WT and *Mtgr1*^−/−^ enteroids, and RNA for all was isolated as described above. For RNA-sequencing studies, mRNA enrichment and cDNA library preparation was performed by the Vanderbilt Technologies for Advanced Genomics (VANTAGE) facility utilizing the Illumina Tru-seq stranded mRNA sample prep kit. Sequencing was performed at Single-Read 50 HT bp on the Illumina HiSeq 2500. After adapter trimming by Cutadapt^23^, RNA-seq reads were aligned to the mm10 genome using STAR^24^ and quantified by featureCounts^25^. Differential analyses were performed by DESeq2^26^, which determined the log2 fold changes, Wald test p-values, and adjusted p-value (FDR) by the Benjamini-Hochberg procedure. The significantly changed genes were assessed with an FDR < 0.05. Gene set enrichment analysis (GSEA) was performed using GSEA software version 4.03 maintained by the Broad Institute^27,28^.

### Single Cell RNA-sequencing

Mouse ileal tissues were used to generate single-cell RNA-seq data, following a methodology similar to previous studies^29,30^. In brief, mouse tissues were incubated in a chelating buffer composed of 20mM HEPES and 3mM EDTA in DPBS for 1.25hrs. Tissue was then transferred to 10ml of PBS and shook vigorously for 2–3 minutes to liberate crypts, which were then passed through a 70μm filter and washed by centrifugation at 300*g* for 5 minutes and resuspension in PBS. Isolated crypts were pelleted and resuspended in a solution of cold active protease (5 mg/ml Protease from *Bacillus licheniformis* and 2.5 mg/mL Dnase in PBS) and incubated for 25 minutes at 4°C. Following this, the tissues were gently pipetted 10–20 times to obtain single cells and passed through a 70μm filter into a clean tube. Isolated cells were pelleted by centrifugation at 700g for 5 min and washed in PBS containing 0.02% BSA three times before final resuspension in PBS containing 15% Optiprep and filtered using a 40μm flowmi filter. The resulting cell suspensions underwent filtration, washing, and quality inspection before being loaded onto inDrops for microfluidic capture. The inDrops scRNA-seq procedure was carried out according to a modified protocol^31,32^. Single-cell libraries were prepared for sequencing as detailed in previous documentation^33^. These libraries, each containing an estimated 2000–3000 cell transcriptomes, were then sequenced on the Novaseq6000 platform, generating approximately 125 million reads per library. Data were processed according to an established pipeline^34^ and filtered using dropkick^35^.

### Electron Microscopy

Specimens were processed for transmission electron microscopy (TEM) and imaged in the Vanderbilt Cell Imaging Shared Resource: Research Electron Microscopy facility. Briefly, enteroid-containing Matrigel plugs were fixed in 2.5% glutaraldehyde in 0.1M cacodylate buffer, pH7.4 at room temperature (RT) for 1 hour, then transferred to 4°C overnight. The samples were washed in 0.1M cacodylate buffer, incubated for 1 hour in 1% osmium tetraoxide at RT, and washed with 0.1M cacodylate buffer. The samples were then dehydrated through a graded ethanol series followed by 3 exchanges of 100% ethanol. Next, the samples were incubated for 5 minutes in 100% ethanol and propylene oxide (PO) followed by 2 exchanges of pure PO. Samples were then infiltrated with 25% Epon 812 resin and 75% PO for 30 minutes at RT. Next, they were infiltrated with Epon 812 resin and PO [1:1] for 1 hour at RT, then overnight at RT. The next day, the samples went through a [3:1] (resin: PO) exchange for 3–4 hours and were incubated with pure epoxy resin overnight. Samples were then incubated in 2 more changes of pure epoxy resin and allowed to polymerize at 60°C for 48 hours.

500–1000 nm-thick sections were cut for ultra-structure identification. Then, 70–80 nm ultra-thin sections were cut from the region of interest, collected on 300-mesh copper grids, and post-stained with 2% uranyl acetate followed by Reynold’s lead citrate. Samples were subsequently imaged on the Philips/FEI Tecnai T12 electron microscope.

### Live cell imaging

Crypts were plated according to standard protocols and immediately placed in the incubation chamber of the EVOS^®^ FL Auto Cell Imaging System. Organoids were imaged every 15 minutes over 5 days with periodic location updates to maintain image focus.

### Statistics

Unless noted, statistical analysis was performed in Graphpad Prism 8 Software using Student’s t-test (unpaired, two-tailed) for single timepoints, or two-way ANOVA and Sidak’s multiple comparison post-test for time course analyses. Samples were excluded if determined to be statistical outliers based on “robust regression and outlier removal” (ROUT) analysis. For all studies, center values represent experimental mean, error is represented by standard error of the mean, and *P* < 0.05 is considered significant.

## Results

### MTGR1 is widely expressed in the intestine.

**Figure 1:**
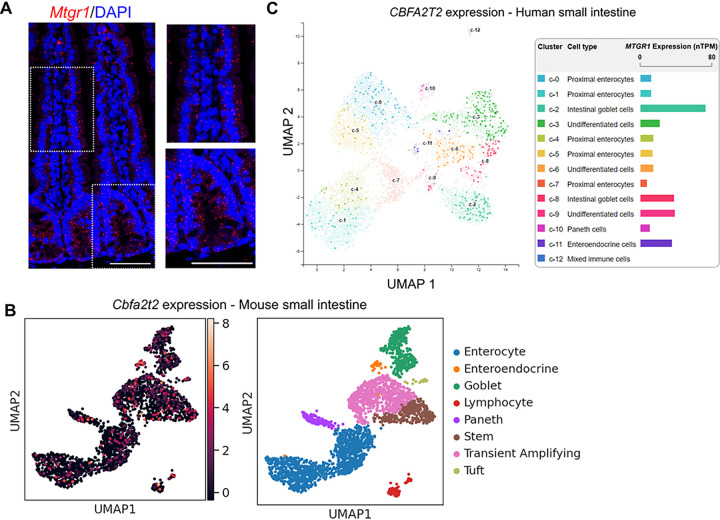
MTGR1 is widely expressed in the small intestine. **(A)** *Mtgr1* mRNA assessed by RNAScope in the WT small intestine. Results representative of 3 independent experiments. Dotted lines indicate inset areas. Scale bar = 100μm. **(B)** Uniform manifold approximation and projections (UMAPs) showing *Mtgr1 (Cbfa2t2)* expression in the murine jejunum by scRNA-sequencing (left) and associated cell clusters (right). n=2 mice. **(C)** Human *MTGR1 (CBFA2T2)* expression was queried from the Human Protein Atlas scRNA-sequencing data. *MTGR1*expression is visualized by UMAP (left) and bar graphs (right) in various intestinal cell types.

As multiple epithelial cell types exist in the small intestine, cellular expression patterns can be used to infer genetic contributions to cell type-specific functions. To define MTGR1 expression patterns within the intestinal crypt, we first utilized *in situ* staining methods to spatially visualize *Mtgr1* transcripts in the murine small intestine (Fig. 1A). As previously reported, *Mtgr1* expression was dispersed throughout the intestinal crypt and did not appear to be specifically localized to distinct cell populations^16^. As *in situ* staining does not easily allow for overlay with multiple cell lineage markers, we next investigated *Mtgr1* expression in specific cell types via single-cell RNA-sequencing (scRNA-seq) of the murine ileum^29,36^. These results confirm widespread *Mtgr1* expression in various intestinal cells that was not restricted to specific cellular lineages (Fig. 1B and **Supplemental Fig. 1**). Similar results were observed in the human small intestine (Fig. 1C). Here, we queried publicly available scRNA-seq data from the Human Protein Atlas (GSE125970), and again, MTGR1 expression was observed in multiple differentiated and undifferentiated cell types^37,38^. Thus, *Mtgr1* is widely expressed throughout the intestinal epithelium.

### MTGR1 loss dysregulates ISC populations in vivo.

**Figure 2:**
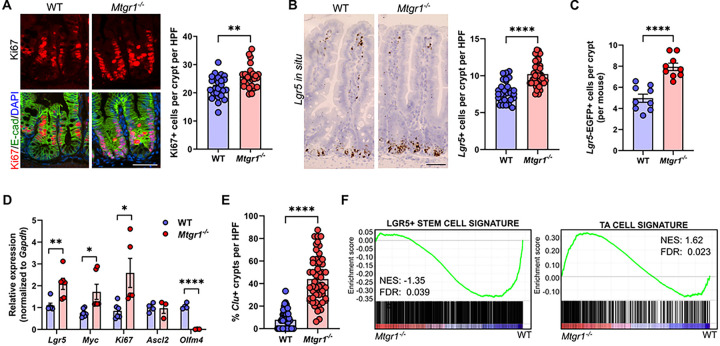
MTGR1 loss deregulates intestinal stem cell programs and promotes transit amplifying identity. **(A)** Immunofluorescent staining for Ki67 (red), E-cadherin (green), and nuclei (DAPI, blue) in the small intestine of 8–12-week-old WT and *Mtgr1*^*−/−*^ mice. n=4 mice per genotype, >20 high-powered fields (HPFs) er mouse. **(B)** *Lgr5* mRNA expression was visualized in the WT and *Mtgr1*^*−/−*^ small intestine by RNAscope. N = 3–4 mice per genotype, 12 HPFs per mouse. **(C)** *Mtgr1*^*−/−*^ mice were intercrossed with the *Lgr5-cre-EGFP* reporter strain and *Lgr5-EGFP* was assessed by immunofluorescence. Quantification shows the number of GFP positive cells in each reporter positive crypt, per mouse. N = 9 mice per genotype. **(D)** qPCR of stem cell markers *Lgr5, Myc, Ki67, Ascl2*, and *Olfm4* in intestinal crypt isolates from *Mtgr1*^*−/−*^ and WT mice (n = 3–6 mice per genotype). Results were normalized to *Gapdh* and represented as fold change over WT expression. **(E)** Clusterin *(Clu)* mRNA expression was visualized in the WT and Mtgr1^−/−^ small intestine by RNAscope. Quantification shows the percent of crypts with *Clu* expression per high powered field. n = 2 mice per genotype, > 20 HPFs per mouse. **(F)** Crypts were collected from WT and *Mtgr1*^*−/−*^ mice and assessed by RNA-sequencing. Gene set enrichment analysis was performed for gene sets associated with *Lgr5*+ stem cells (left) and transit amplifying cell (TA, left) populations. NES = normalized enrichment score. *P<0.05, **P<0.01, ****P<0.0001, Student’s t test (A-E), significance indicated by FDR q value (F). Scale bars = 100μm.

Next, we directly investigated the function of MTGR1 in ISC biology. Interestingly, higher levels of cellular proliferation have been reported in mice globally lacking MTGR1 (*Mtgr1*^−/−^), which may be due to loss of MTGR1-mediated downregulation of Wnt pathway genes via TCF4 repression^15,19,39^. Here, we first confirmed the expansion of proliferative cells in the crypts of *Mtgr1*^−/−^ mice via quantifying Ki67 expression (Fig. 2A). We next hypothesized that this increase in proliferation may be associated with higher numbers of LGR5 + ISCs, due to their role in maintaining proliferation in the intestine at baseline^15,19,39^. *Lgr5*-expressing cells in the small intestine were identified by *in situ* hybridization. Here, we determined that *Mtgr1*^−/−^ mice indeed had higher numbers of *Lgr5* + cells per crypt as compared to WT mice (Fig. 2B). This was further confirmed by intercross of *Mtgr1*^−/−^ mice with the *Lgr5-Cre-EGFP* reporter line (Fig. 2C), which expresses EGFP from the *Lgr5* locus. Analysis of these mice again showed increased numbers of *Lgr5*-EGFP + cells in *Mtgr1*^−/−^ versus WT *Lgr5-Cre-EGFP* mice. Higher levels of *Lgr5*, *Ki67*, and the Wnt target transcript *Myc* were also observed in *Mtgr1*^−/−^ crypts by qPCR (Fig. 2D). Based on these results, it seems likely that the LGR5 + CBC population and overall ISC function may increase when MTGR1 is lost.

While *Lgr5* is often regarded as the canonical identifier of CBCs and a robust ISC marker, we next assayed for expression of *Ascl2* and *Olfm4*, which are highly expressed in CBCs along with *Lgr5*^40,41^ (Fig. 2D). Interestingly, although *Lgr5* transcript and *Lgr5*-EGFP + cells were consistently increased with MTGR1 loss, neither *Ascl2* nor *Olfm4* mirrored these changes. Here, we observed that *Ascl2* transcript remained unchanged in *Mtgr1*^−/−^ crypts, while *Olfm4* was nearly undetectable in *Mtgr1*^−/−^ crypts. Similarly, survey of markers associated with non-CBC ISC populations revealed further differences, such as greater numbers of *Clusterin* (*Clu*)-expressing cells (Fig. 2E)^6^.

As cell identity and function are complex, we next isolated WT and *Mtgr1*^−/−^ crypts and expanded our transcriptomic analysis of MTGR1-dependent changes in intestinal cell types through bulk RNA sequencing. Differential expression data was then analyzed by gene set enrichment analysis (GSEA), and results from WT and *Mtgr1*^−/−^ crypts were compared to gene sets established from prototypic *Lgr5*-expressing ISCs (Fig. 2F and **Supplemental Fig. 2**)^9^. Despite increased expression of *Lgr5* itself in the setting of MTGR1 loss, total canonical *Lgr5* + ISC gene signatures were significantly de-enriched in crypts collected from *Mtgr1*^−/−^ mice. Instead, *Mtgr1*^−/−^ samples were enriched for genes which indicated an expansion of TA populations, which is also consistent with increased proliferation noted in *Mtgr1*^−/−^ crypts (Fig. 2A). Taken together, these results indicate that while more cells in *Mtgr1*^−/−^ crypts express *Lgr5*, these cells may instead resemble more differentiated TA cells, rather than true *Lgr5* + ISCs.

### MTGR1 is required for enteroid viability.

**Figure 3:**
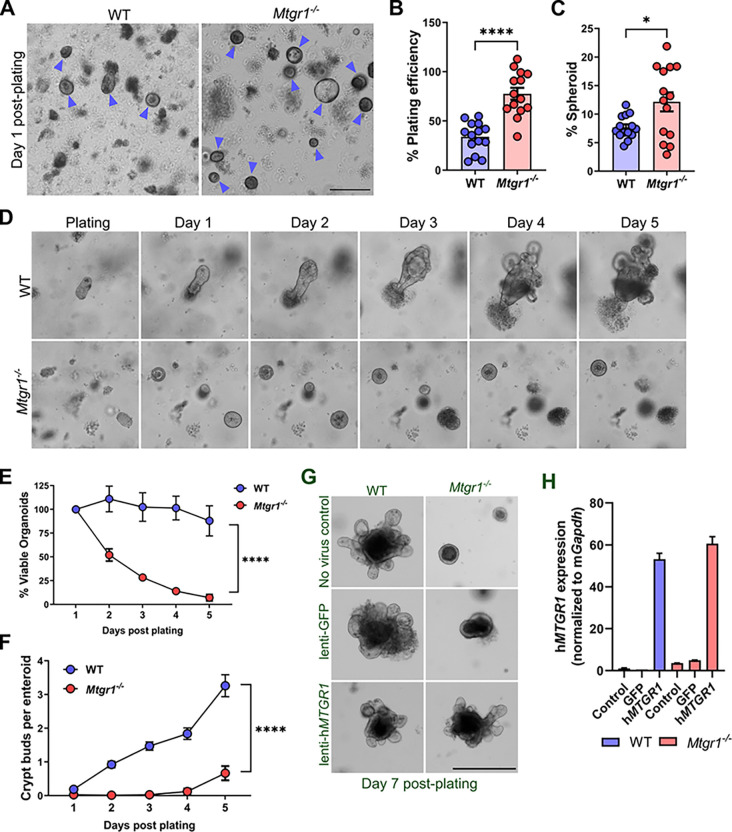
MTGR1 loss increases initial plating efficiency but MTGR1 is required for enteroid survival. **(A)** Crypts were isolated from WT and *Mtgr1*^*−/−*^ mice and plated as intestinal enteroids. Representative images of organoids at day 1 post-plating with enteroids marked by blue arrows. Scale bar = 200μm (left), representative of 4 independent experiments. **(B)** Quantification of overall plating efficiency (enteroids established divided by crypts plated) and **(C)** percentage of enteroids with cystic morphology calculated per well at day 1 post-plating. n = 14 wells per genotype. **(B)** 5-day timelapse imaging of WT and *Mtgr1*^*−/−*^ enteroids. **(E)** Average enteroid viability post-plating, shown as the percent viable enteroids remaining from day 1. N ≥ 12 wells per *genotype*. **(F)** Quantification of crypt budding post-plating. n > 65 organoids per genotype for early timepoints (day 1–3), n > 5 for later timepoints (day 4–5). **(G)** WT and *Mtgr1*^*−/−*^ crypts were transduced with lentiviral GFP or human *MTGR1* and plated to allow organoid formation. Representative images at day 7 post-infection/plating. Scale bars = 500μm. Transduced enteroids were collected at day 7 post-infection for mRNA analysis of human *MTGR1*. Results were normalized to *Gapdh* and shown as fold change over WT non-transduced controls. *P<0.05, ****P<0.0001, Student’s t test (B-C), two-way ANOVA (E-F).

In our *in vivo* analysis, loss of MTGR1 appeared to deregulate ISC-associated genes and promote TA-associated differentiation. Thus, we questioned whether *Mtgr1*^−/−^ ISCs were fully functional as multipotent intestinal stem cells. We next specifically tested whether MTGR1 loss affected overall ISC function using the small intestinal organoid or “enteroid” system. Since enteroids rely on ISCs for their establishment and growth, enteroid formation efficiency can be used to assess general stem cell function and fitness^3^. Here, enteroids were established from duodenal crypts harvested from WT and *Mtgr1*^−/−^ mice, and enteroid formation efficiency was assessed after 24 hours in culture (Figs. 3A and 3B). By dividing the number of enteroids formed by the number of crypts plated, we noted an approximately 2-fold enhancement of enteroid formation in the setting of MTGR1 loss. We also observed higher percentages of *Mtgr1*^−/−^ enteroids with a cystic, spheroid morphology (Fig. 3C), a phenotype associated with increased Wnt tone^20^ ,compared to WT enteroids. Taken together, these results suggest that MTGR1-deficient ISCs are indeed initially functionally competent, despite de-enrichment of a canonical *Lgr5* transcriptional profile.

While initial enteroid formation was augmented in *Mtgr1*^−/−^ cultures, we observed striking viability defects in *Mtgr1*^−/−^ enteroids within 48 hours of initial plating. Daily imaging (Fig. 3D) and viable enteroid counts (Fig. 3E) revealed that *Mtgr1*^−/−^ cultures failed almost completely by day 5 post-plating. While WT enteroids formed crypt buds by day 3, *Mtgr1*^−/−^ enteroids rarely developed crypt buds, even in the structures that survived until day 5 (Fig. 3F). These findings were also confirmed by live cell imaging, which showed no morphological changes in *Mtgr1*^−/−^ enteroids throughout the 5-day period, until organoid death (**Supplemental videos SV1 and SV2**). Importantly, restoration of *MTGR1* expression via lentiviral transduction rescued *Mtgr1*^−/−^ enteroids and restored branching morphology, confirming the MTGR1 dependency of this phenotype (Fig. 3G and 3H). Thus, MTGR1 appears to be required for *ex vivo* enteroid survival and expansion.

### Inhibition of programmed cell death does not rescue Mtgr1 ^−/−^ viability.

**Figure 4:**
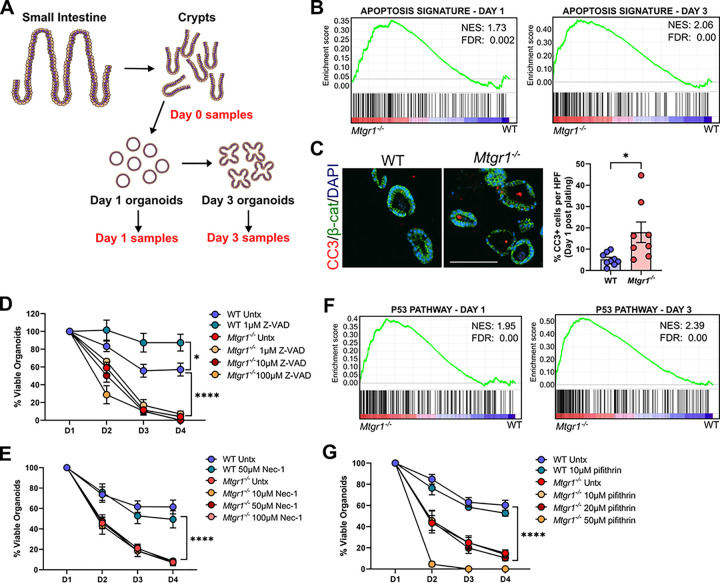
MTGR1 loss increases enteroid apoptosis, but effects cannot be rescued by apoptosis inhibition. **(A)** Schematic of RNA-sequencing experiment of crypts and enteroids. n = 3 mice per genotype. **(B)** GSEA of “Hallmark” collection apoptosis-related genes in *Mtgr1*^*−/−*^ enteroids at day 1 (left) and day 3 (right) post-plating. NES = normalized enrichment score. **(C)** Enteroids were fixed and embedded at day 1 post-plating, and apoptotic cells were marked by immunofluorescent staining against cleaved caspase-3 (CC3, red). β-catenin (green) and DAPI (blue) were used for co-staining. Quantification shown as percent CC3-positive cells per high powered field (HPF). Scale bar = 200μm. n = 8–9 HPFs per genotype. **(D)** Enteroids were plated and overlaid with media containing indicated concentrations of the caspase inhibitor, Z-VAD-FMK, or **(E)** the necrosis inhibitor, necrostatin. Enteroids were counted daily and normalized to day 1 numbers. n ≥ 6 wells per condition. **(F)** GSEA of “Hallmark” collection p53 pathway-related genes in *Mtgr1*^*−/−*^ enteroids at day 1 (left) and day 3 (right) post-plating. **(G)** Enteroids were plated and overlaid with media containing the p53 inhibitor, pifithrin, as indicated. n ≥ 3 wells per condition. *P<0.05, ****P<0.0001, Student’s t test (C) or two-way ANOVA (D, E, G), significance indicated by FDR q value (B, F).

We next aimed to determine the mechanism driving the viability loss in *Mtgr1*^−/−^ enteroids. As MTGR1 is a transcriptional co-repressor, we again utilized a bulk RNA-sequencing approach to broadly investigate MTGR1-dependent changes in gene expression. Briefly, crypts were isolated from age-matched WT and *Mtgr1*^−/−^ mice, and mRNA was collected at the time of crypt isolation (day 0), or at day 1 and day 3 post-plating to yield matched RNA sets of crypts, day 1 enteroids, and day 3 enteroids (Fig. 4A). After RNA-sequencing, differential expression profiles were generated and analyzed using GSEA^27,28^.

Due to the rapid loss of established cultures, we hypothesized that MTGR1 loss may aberrantly activate programed cell death pathways to drive the observed decline in enteroid viability. Indeed, GSEA analysis from the Hallmark gene set collection identified a significant enrichment in apoptosis-associated genes in *Mtgr1*^−/−^ enteroids at both day 1 and day 3 post-plating (Fig. 4B). *Mtgr1*^−/−^ enteroids collected at day 1 post-plating also displayed higher numbers of apoptotic cells as compared to WT enteroids (Fig. 4C) as measured by fluorescent immunohistochemistry (**IHC**) against cleaved caspase-3. However, inhibiting apoptosis using the cell-permeable pan-caspase inhibitor, Z-VAD-FMK, failed to improve survival of *Mtgr1*^−/−^ enteroids (Fig. 4D), even at concentrations which improved viability in WT cultures^42^. Likewise, inhibition of necroptosis, whose dysregulation has been noted in intestinal inflammatory diseases^43–45^, had no effect on *Mtgr1*^−/−^ enteroid viability (Fig. 4E). Finally, we assessed the impact of p53 inhibition, as p53-related gene sets were also positively enriched in *Mtgr1*^−/−^ samples by GSEA (Fig. 4F). As with Z-VAD-FMK, inhibition of p53-dependent apoptosis with pifithrin-α had no effect on *Mtgr1*^−/−^ enteroid survival (Fig. 4G)^46^. Thus, despite increases in apoptosis, inhibition of known cell death mechanisms is insufficient to rescue *Mtgr1*^−/−^ enteroid viability.

### Proliferation and ISCs are lost in MTGR1-deficient enteroids.

**Figure 5:**
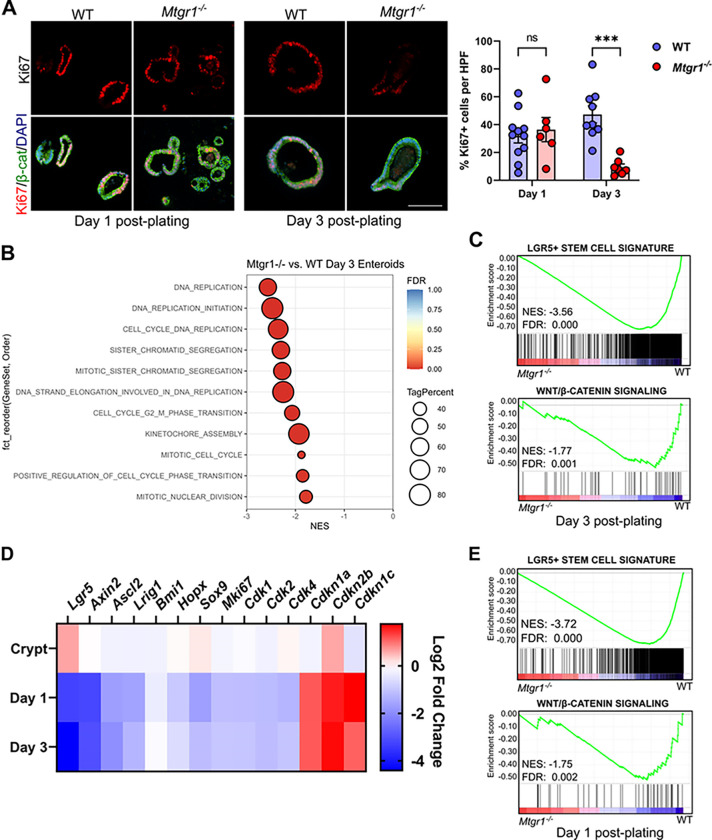
MTGR1 loss decreases proliferation and stem cell-associated gene expression during organoid maturation. **(A)** WT and *Mtgr1*^*−/−*^ enteroids were fixed at day 1 and day 3 post-plating and proliferative cells were marked via immunohistochemistry for Ki67 (red). β-catenin (green) and DAPI (blue) were used for co-staining. Quantification shown as percent Ki67-positive cells per high powered field (HPF). Scale bar = 200μm. n≥7 fields per genotype per timepoint. **(B)** Gene set enrichment analysis (GSEA) of day 3 RNA-sequencing results using cell cycle-related gene sets queried from the Gene Ontology collection. NES = normalized enrichment score. Tag % = the percentage of gene hits before (for positive ES) or after (for negative ES) the peak in the running ES, indicating the percentage of genes contributing to the ES. **(C)** GSEA of day 3 RNA-sequencing results with intestinal stem cell- (top) and Wnt-associated (bottom) gene sets. **(D)** Heatmap of RNA-sequencing results of stem cell, cyclin dependent kinases, and cyclin-dependent kinase inhibitors from crypt, day 1, and day 3 results. Represented as the Log2 fold change of *Mtgr1*^*−/−*^ results as compared to WT at that timepoint. **(E)** GSEA of day 1 RNA-sequencing results with intestinal stem cell- (top) and Wnt-associated (bottom) gene sets. ns = nonsignificant, ***P<0.001, Student’s t test (A), significance indicated by FDR q value (B, C, E).

Due to constant cell clearance, actively cycling stem cells and high levels of proliferation are necessary to maintain intestinal cell populations^47^. Thus, rather than aberrant apoptosis, we next hypothesized that the viability defect in *Mtgr1*^−/−^ enteroids may instead be due to reduced proliferation and/or depletion of ISCs. To determine cell proliferation, sections from enteroids embedded at day 1 and day 3 post-plating were assessed by Ki67 IHC (Fig. 5A). Although we observed similar numbers of proliferating cells in day 1 enteroids, by day 3, the enteroid cultures established from *Mtgr1*^−/−^ mice displayed a drastic, nearly 80% reduction in Ki67 + cells. Cell cycle- and proliferation-associated genes were also highly downregulated in *Mtgr1*^−/−^ enteroids by day 3 (Fig. 5B), as well as ISC-associated genes and signaling pathways (Fig. 5C), as determined by GSEA. Interestingly, while numbers of Ki67 + cells were similar between WT and *Mtgr1*^*−/−*^ enteroids at day 1 post-plating, proliferation-, ISC-, and Wnt-associated genes were still significantly downregulated at this early timepoint (Fig. 5D, 5E, and **Supplemental Table S3**). *Mtgr1*^−/−^ enteroids, at either day 1 or day 3 post-plating, also demonstrated significant upregulation of the cell cycle inhibitors *Cdkn1a, Cdkn1c, Cdkn2b*. These results indicate that viability defects in *Mtgr1*^−/−^ enteroids may arise from proliferation defects and the inability to maintain cycling ISC populations *ex vivo*.

### MTGR1 loss promotes absorptive enterocyte differentiation.

**Figure 6:**
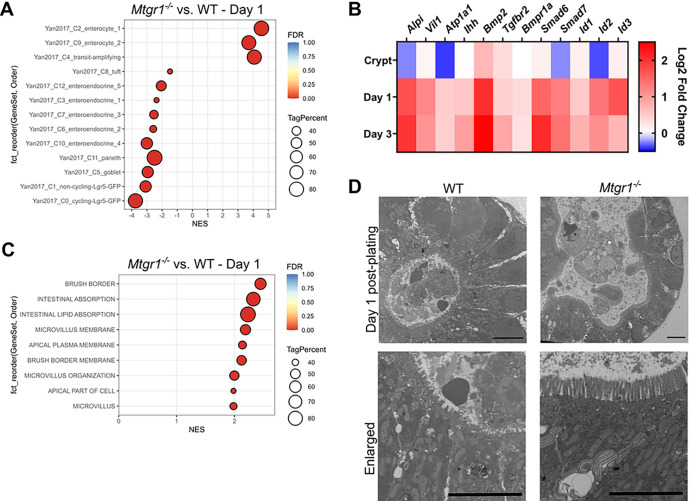
MTGR1 loss promotes absorptive enterocyte differentiation. **(A)** Gene set enrichment analysis (GSEA) of *Mtgr1*^*−/−*^ day 1 organoid RNA-sequencing results using gene sets representing intestinal epithelial cell types. NES = normalized enrichment score. Tag % = the percentage of gene hits before (for positive ES) or after (for negative ES) the peak in the running ES, indicating the percentage of genes contributing to the ES. Significance indicated by FDR q value. **(B)** Heatmap of RNA-sequencing results of enterocyte- and differentiation-associated genes in crypts, day 1 enteroids, and day 3 enteroids. Represented as the log2 fold change of *Mtgr1*^*−/−*^ results as compared to WT at that timepoint. **(C)** GSEA of day 1 *Mtgr1*^*−/−*^ RNA-sequencing results using gene sets representing microvilli and brush border biology queried from the Gene Ontology collection. **(D)** Representative electron microscopy images from WT and *Mtgr1*^*−/−*^ day 1 enteroid samples. Scale bar = 5μm.

After expansion in the TA zone and exit from the intestinal crypt, most ISC-derived cells rapidly undergo differentiation into non-proliferative cell lineages^48^. As stem and proliferative cell populations are not maintained in *Mtgr1*^−/−^ enteroids, and their loss appears unlikely due to programed cell death, we hypothesized that the loss of enteroid viability may be due to augmented ISC differentiation into non-proliferative cells. Indeed, this would result in failed ISC amplification, inability to maintain enteroid cultures, and eventual death of terminally differentiated cells. Therefore, we next broadly surveyed differentiated intestinal cell types using GSEA. In agreement with the data presented in Fig. 5 and the results from intestinal crypts (Fig. 2F), cycling *Lgr5* + cells again appeared to be greatly depleted, and were the most reduced cell population in *Mtgr1*^−/−^ enteroids at both day 1 and day 3 post-plating (Fig. 6A and **Supplemental Fig. 3**). Conversely, more differentiated TA populations were greatly increased in *Mtgr1*^−/−^ enteroids, as were fully differentiated populations of enterocytes.

To more clearly define whether MTGR1 loss indeed accelerates intestinal differentiation, we next determined the expression of genes associated with villi and enterocyte differentiation pathways. We first surveyed classical markers of the enterocyte lineage, such as intestinal alkaline phosphatase (*Alpi*), as well as genes associated with BMP and IHH pathways, as these work in opposition to Wnt-mediated signaling in order to promote differentiation^3^. In nearly all cases, these differentiation-associated genes were significantly enriched in *Mtgr1*^−/−^ enteroids as compared to matched WT samples (Fig. 6B and **Supplemental Table S4**). GSEA analysis also determined significant enrichment of genes associated with features of absorptive enterocytes, such as the brush border, microvilli, and intestinal absorption (Fig. 6C). Finally, the cellular structure of day 1 enteroids was investigated using transmission electron microscopy (TEM). Comparison of WT and *Mtgr1*^−/−^ enteroids illustrates an expansion of the apical cell surface as well as more pronounced and mature microvilli (Fig. 6D). Altogether, these results indicate that *Mtgr1*^−/−^ ISCs are likely further differentiated at baseline than WT ISCs, and upon *ex vivo* culture, differentiation is so accelerated that proliferative cells are lost entirely to the absorptive enterocyte lineage, to the point that cultures cannot be maintained.

### Secretory cells promote the survival of Mtgr1 ^−/−^ enteroids.

**Figure 7:**
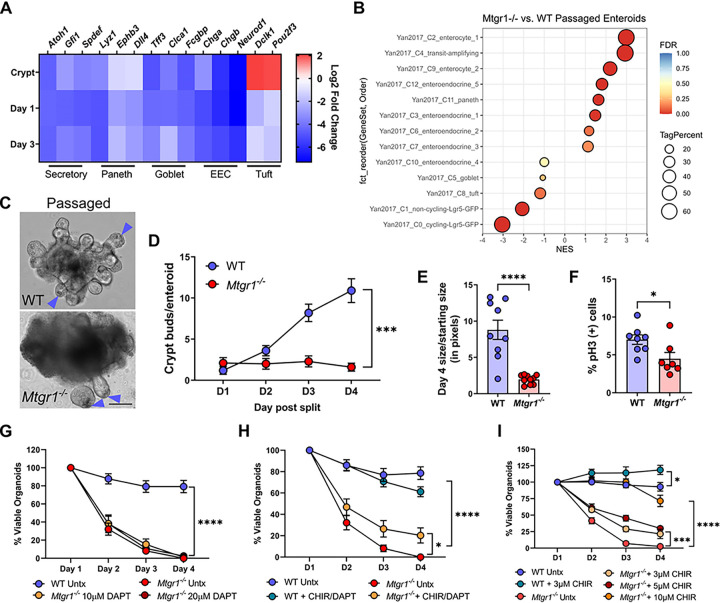
High-WNT, but not secretory differentiation, rescues Mtgr1 null enteroids. **(A)** Heatmap of RNA-sequencing results of secretory-associated genes from crypts, day 1 enteroids, and day 3 enteroids. Results represented as the log2 fold change of *Mtgr1*^*−/−*^ samples as compared to WT at that timepoint. **(B)** Gene set enrichment analysis (GSEA) of passaged *Mtgr1*^*−/−*^ enteroids using gene sets representing intestinal epithelial cell types. NES = normalized enrichment score. Tag % = the percentage of gene hits before (for positive ES) or after (for negative ES) the peak in the running ES, indicating the percentage of genes contributing to the ES. Significance indicated by FDR q value. **(C)**Representative images showing passaged WT enteroids and *Mtgr1*^*−/−*^ enteroids. Passaged WT and *Mtgr1*^*−/−*^ enteroids both had discernible Paneth cells in the crypt base (arrows). Scale bar = 200μm. (**D)** Quantification of crypt buds per Passaged enteroids post-split, n=10 enteroids per genotype. **(E)** Passaged enteroids were imaged at day 1 and day 4 post-passage and enteroid area measured via ImageJ. Change in size was calculated by dividing day 4 measurements by those taken at day 1. n = 10 enteroids per genotype **(F)** Passaged *Mtgr1*^−/−^ enteroids were fixed and stained with phospho-histone H3 (pH3) to mark proliferative cells. Quantification shown as percent pH3-positive cells per high powered field (HPF). n ≥ 7 fields per genotype. **(G).** Enteroids were plated and overlaid with media containing indicated concentrations of the gamma secretase inhibitor, DAPT. Enteroids were counted daily and normalized to day 1 numbers. n = 7–8 wells per condition. **(H)** WT and *Mtgr1*^*−/−*^ enteroids were plated and supplemented with 3μM of CHIR-99021 (CHIR) and 10μM DAPT. Enteroids were counted daily and normalized to day 1 numbers. n = 8 wells per condition. (I) WT and *Mtgr1*^*−/−*^ enteroids were plated with CHIR as indicated. Enteroid numbers were assessed daily and normalized to day 1 results. n ≥ 4 wells per condition. *P<0.05, ***P<0.001, ****P<0.0001, significance indicated by FDR q value (A, B), two-way ANOVA (D, G, H, I), and Student’s t test (E, F).

Previous research has indicated that MTGR1 is necessary for the differentiation of multiple secretory lineages in the small intestine, including Paneth, goblet, and enteroendocrine cells (EECs)^15,18^. Thus, we next confirmed the loss of these cell types in *Mtgr1*^−/−^ crypts and enteroid cultures. A query of RNA-seq results indeed showed downregulation of genes associated with general secretory cell differentiation and those enriched in the Paneth, goblet, and EEC lineages (Fig. 7A, **Supplemental Fig. 2**, and **Supplemental Table S5**). Interestingly, loss of MTGR1 had the opposite effect on the tuft cell lineage *in vivo*, which was increased in the intestinal crypts; however, this same cell population appeared to be downregulated in *Mtgr1*^−/−^ enteroids. Together, these results further define the variable effect of MTGR1 on intestinal cell differentiation.

Secretory lineages, specifically Paneth cells, are crucial regulators of CBCs and provide Wnt ligands that maintain ISC stemness, and crypts from mice lacking Paneth cells cannot form enteroid cultures without Wnt supplementation^49–52^. Thus, the loss of *Mtgr1*^−/−^ CBCs, and by extension enteroid cultures, may be due to the lack of Paneth cells. While the majority of enteroids from *Mtgr1*^−/−^ mice died by day 5, a small number of surviving enteroids occasionally could be maintained and passaged. We hypothesized that these few “passaged” *Mtgr1*^−/−^ enteroids may provide clarity regarding mechanisms that could rescue MTGR1-dependent growth defects. To this end, we established and analyzed two independent *Mtgr1*^−/−^ enteroid lines by RNA-sequencing to determine how these surviving cells differed from *Mtgr1*^−/−^ cultures that subsequently died. Cell type analysis via GSEA found no deficiencies in Paneth or EEC populations in passaged *Mtgr1*^−/−^ enteroids in comparison to WT enteroids (Fig. 7B), and Paneth cells could often be distinguished in the crypt base (Fig. 7C). Therefore, Paneth cells may drive the survival of passaged *Mtgr1*^−/−^ cultures. However, despite the presence of Paneth cells, passaged *Mtgr1*^−/−^ enteroids still displayed striking alterations in morphology, an inability to form enteroid buds (Fig. 7D), and little expansion in size over time (Fig. 7E). As observed in early enteroids at day 1 and day 3 post-plating, passaged *Mtgr1*^−/−^ enteroids also showed significant reductions in ISC populations by GSEA (Fig. 7B) and proliferation by immunofluorescent IHC against the proliferative marker phospho-histone H3 (pH3, Fig. 7F). Thus, while Paneth cells likely aid the survival of passaged *Mtgr1*^−/−^ enteroids, these results suggest that the presence of Paneth cells alone may not be sufficient to rescue growth, morphology, or ISC populations in the setting of MTGR1 loss.

### Mtgr1 ^−/−^ enteroids can be rescued by high Wnt activity.

As our investigation of passaged *Mtgr1*^−/−^ enteroid lines showed that Paneth cells may assist ISC survival in the context of MTGR1 loss, we next employed various combinations of small molecules to promote secretory cell differentiation and survival of *Mtgr1*^−/−^ ISCs. First, we utilized DAPT, a γ-secretase inhibitor that has been shown to increase secretory cell numbers *ex vivo* at the expense of absorptive lineages. However, γ-secretase inhibitor treatment failed to rescue *Mtgr1*^−/−^ enteroid growth *ex vivo* (Fig. 7G), even when treatment was begun *in vivo* prior to and continuing through enteroid establishment (data not shown). Next, we combined DAPT with the Wnt pathway agonist, CHIR 99021, as this combination should greatly promote Paneth cell differentiation^53^. These studies revealed only a modest induction of enteroid survival (Fig. 7H). Finally, we investigated whether Wnt pathway activation alone was sufficient to maintain *Mtgr1*^−/−^ ISCs. Surprisingly, low-dose CHIR 99021 treatment (3μM), a concentration sufficient to promote WT ISCs and mimic the effects of recombinant Wnt3a, only had a modest effect on *Mtgr1*^−/−^ enteroid survival (Fig. 7I)^54^. This could be overcome by increasing the concentration of CHIR 99021 (10 μM) to sustain survival and growth of *Mtgr1*^−/−^ ISCs *ex vivo*. All together, these results indicate that while MTGR1 loss increases cell proliferation and *Lgr5* expression, the overall functionality and differentiation state of *Mtgr1*^−/−^ ISCs is severely compromised. While previously regarded as a transcription factor regulating secretory/absorptive lineage specification, these data indicate that MTGR1 is necessary for maintaining overall dedifferentiation and stem cell status in the small intestine.

## Discussion

In this study, we establish that MTGR1 is widely expressed in the intestine and is required for the proper function of ISCs. While MTGR1 loss led to increased proliferation and *Lgr5* + stem cell numbers, a prototypic *Lgr5 +* cell transcriptional profile was absent. Instead, our data indicate that *Mtgr1*^−/−^ ISCs were more fully differentiated and may not wholly function as bona-fide stem cells. In support of this, expression of *Clusterin*, an ISC-associated gene normally indicative of injury response and loss of *Lgr5* + CBCs, was increased, as were genes associated with the small intestinal TA compartment. Cells from MTGR1-deficient crypts also demonstrated defective stem cell faculty, as they failed to proliferate in *ex vivo* enteroid cultures in association with increased apoptosis, cell cycle arrest, aberrant absorptive lineage differentiation, and loss of *Lgr5* + ISCs as noted by transcriptomic profiling. Indeed, survival of *Mtgr1*^−/−^ enteroids was possible through high Wnt pathway activity, indicating that strong stem cell-promoting signals correct the intrinsic defects of MTGR1 deficiency. Thus, MTGR1 loss significantly impacted survival and differentiation of multiple intestinal cell types, identifying a novel and crucial role of this gene in ISC function.

MTGR1 belongs to the Myeloid Translocation Gene family, member of which have been widely implicated in stem cell maintenance and lineage commitment in the hematopoietic system and beyond. In the homeostatic intestine, *Mtg16*^−/−^ and *Mtgr1*^−/−^ mice both display increased proliferation and altered secretory lineage allocation^15,16,18,39,55,56^. However, while the MTG family members share significant sequence homology and generally similar phenotypes, they can also exhibit diverse effects in the gut, particularly in specific cell types. For example, while loss of MTGR1 depleted nearly all intestinal secretory cells, loss of MTG16 appeared to regulate more subtle cell fate decisions between goblet cells and EECs. Meanwhile, ISC-specific roles have been investigated for some, but not all MTG family members. In the small intestine, it has recently been reported that MTG16 and MTG8 are both enriched in + 4/+5 cells, where they repress ISC-specific genes to control exit from the stem cell niche^16^. On the other hand, enrichment of MTGR1 was not observed in ISCs, which instead was broadly expressed throughout the intestinal crypt. Both *Mtg8* and *Mtg16* were also reported to be negatively regulated by the ISC-associated Notch signaling pathway; however, Notch activity did not appear to similarly regulate *Mtgr1*^16^. Finally, unlike the results described herein from *Mtgr1*^−/−^ crypts, neither loss of *Mtg8* nor *Mtg16* was incompatible with enteroid culture and ISC maintenance *ex vivo*^16*,*55*,*57^. Thus, each member of the MTG family appears to play crucial, distinct roles in intestinal biology and differentiation. Indeed, our data suggests *Mtgr1* is uniquely required among its family members to maintain small intestinal ISC function.

While single genes may contribute to ISC function, our results also underscore the complexity of various stem cell populations and caution against the use of single genetic markers as an indicator of cell abundance or function. Nor, as others have pointed out, does the expression of ISC markers truly indicate facultative stem cell function, as not all cells positive for ISC markers will demonstrate lineage tracing even in the setting of injury^58^. Here, while *Lgr5* + cells were increased as measured by qRT-PCR, RNAscope, and intercross with *Lgr5-EGFP-IRES-creERT2* mice, ^58^we observed functional *Lgr5* + stem cells to be greatly depleted in the setting of *Mtgr1* loss, by expanding ISC analysis to include a larger and more complex genetic signature. Furthermore, despite heightened expression of *Lgr5*, these cells were not able to maintain self-renewal *ex vivo* and rapidly differentiated into absorptive cells. Thus, true ISC function may be vastly more complicated than implied by their collection of ISC markers, and how the interplay of multiple proteins and signaling pathways contributes to stem cell function remains to be fully understood.

Interestingly, MTGR1 is well-poised to act as a master regulator of intestinal differentiation and lineage allocation. Although MTGs lack enzymatic activity and DNA-binding capability, they act as scaffolds facilitating transcription factor and epigenetic modifier complex assembly. As such, they associate with basic helix-loop-helix (bHLH) transcription factors, including tissue- and cell type-specific bHLH transcription factors and E proteins (E2A, HEB, and E2–2), which coordinate hematopoietic differentiation with cell cycle exit to repress their transcriptional activity. Importantly, genetic specificity is imparted via interactions with transcription factors bound to specific enhancers. Similar to hematopoietic stem cell and differentiation, transcriptional networks also regulate intestinal programs. Of note, studies to date have implicated MTGR1 in two such transcriptional networks known as key regulators of ISC identity and function: Wnt and Notch^3^. Indeed, our previous research has determined that MTGR1 can compete with β-catenin for TCF4 occupancy and, in doing so, suppresses Wnt transcriptional targets^19^. MTGR1 can also suppress Notch targets via interactions with CSL, a key Notch effector^18^. Despite the ability of MTGR1 to repress ISC-related signaling pathways, which one may expect to augment ISC fitness upon MTGR1 loss, we instead noted that MTGR1 deficiency clearly abrogates ISC function. Thus, further investigation of the function of MTGR1 in ISC-related signaling cascades and identification of bona-fide MTGR1 genomic targets, using methodology such as CUT&RUN, ATAC-seq, and scRNA-seq, will be critical in furthering our understanding of how MTGR1 contributes to crypt and intestinal biology.

In conclusion, our studies more fully elucidate the functional contributions of MTGR1 to small intestinal homeostasis and have identified a novel role for MTGR1 in maintaining *Lgr5* + CBC populations and the dedifferentiated state. While loss of MTGR1 increased *Lgr5* expression and LGR5 + cell numbers, *Mtgr1*^−/−^ ISCs displayed widespread dysregulation of ISC programs and were functionally deficient as compared to WT ISCs. Instead, *Mtgr1*^−/−^ ISCs were primed for cell cycle exit and underwent rapid aberrant differentiation into absorptive enterocytes. Together, these findings indicate that MTGR1 is required for stem cell maintenance in the intestinal epithelium.
